# Intraoperative Pachymetry Using Spectral-Domain Optical Coherence Tomography during Accelerated Corneal Collagen Crosslinking

**DOI:** 10.1155/2013/848363

**Published:** 2013-07-25

**Authors:** Vanissa W. S. Chow, Sayantan Biswas, Marco Yu, Victoria W. Y. Wong, Vishal Jhanji

**Affiliations:** ^1^Hong Kong Eye Hospital, Hong Kong; ^2^Department of Ophthalmology and Visual Sciences, The Chinese University of Hong Kong, Hong Kong; ^3^Department of Ophthalmology, Prince of Wales Hospital, Shatin, Hong Kong; ^4^Centre for Eye Research Australia, University of Melbourne, Melbourne, VIC 3002, Australia

## Abstract

*Purpose*. To evaluate the role of spectral-domain optical coherence tomography (SDOCT) to measure corneal thickness during accelerated corneal crosslinking (CXL). *Methods*. Intraoperative pachymetry was performed using SDOCT and ultrasound pachymetry (USP) in 6 eyes of 6 patients with keratoconus. Pachymetry readings were obtained at baseline, after epithelium removal and after 30 minutes of riboflavin instillation. SDOCT measurements of eyes with and without lid speculum during riboflavin instillation were compared. *Results*. There was no statistically significant difference in central corneal thickness (CCT) measurements between SDOCT and USP (*P* > 0.05 for all). A significant decrease in both CCT (*P* = 0.031) and the thinnest corneal thickness (TCT) (*P* = 0.031) was observed during CXL. There was a greater reduction in CCT (38 ± 6%) with the use of lid speculum as compared to the no-speculum eyes (18 ± 9%) (*P* = 0.100). TCT was also reduced by a greater extent with the use of lid speculum (40 ± 5% versus 26 ± 7%; *P* = 0.100). *Conclusion*. SDOCT can be successfully used to measure intraoperative corneal pachymetry during corneal CXL. SDOCT measurements demonstrated corneal thinning intraoperatively during CXL, which was further accentuated by the use of a lid speculum during the procedure.

## 1. Introduction

Corneal collagen crosslinking (CXL) utilizes ultraviolet A (UVA) light and riboflavin as a photosensitizer to induce covalent crosslink bonds between collagen fibres in the corneal stroma, thereby increasing its biomechanical strength and stability [1]. It is now widely used in the management of keratectasia [2], as well as in selected cases of corneal melting, infective keratitis [3], and bullous keratopathy [4]. However, to avoid damaging the corneal endothelium with the standard UVA surface irradiance of 5.4 J/cm^2^, a uniform and calibrated UVA light source together with a minimum corneal thickness of 400 microns has been proposed based on animal studies and theoretical models [5, 6]. In fact, reduced endothelial cell count is found when corneas of less than 400 microns undergo CXL using the Dresden protocol [7]. Permanent corneal haze is also more commonly found in eyes with thin corneas [8], which seems to be prevented by the use of hypoosmolar riboflavin [9]. Moreover, corneal thickness is shown to further decrease during the CXL procedure [10–14], attributable to dehydration from a combination of epithelium debridement, use of lid speculum and the composition of the riboflavin [15]. Consequently, many surgeons now perform intraoperative ultrasound pachymetry (USP) and use alternative protocols to ensure a minimum corneal thickness of 400 microns during UVA irradiation. However, use of USP leaves a certain doubt whether the repeated measurements are taken at the same location. Also, USP cannot locate the thinnest point on the cornea, which is an important parameter with regards to endothelial safety but is commonly displaced in an ectatic cornea. On the other hand, optical coherence tomography is a quick and noncontact method that employs low-coherence interferometry to obtain cross-sectional images and pachymetric mapping of the cornea. The development of spectral-domain optical coherence tomography (SDOCT) allows faster image acquisition and improved spatial resolution, minimizing effect of eye movement during data acquisition and improving repeatability of the measurements [16–20]. In this study, we demonstrated the ease and usefulness of an intraoperative SDOCT machine to monitor corneal thickness during CXL and compare the change in corneal thickness with and without the use of lid speculum during corneal saturation with riboflavin.

## 2. Patients and Methods

### 2.1. Patient Population

In this prospective study, 6 eyes of 6 consecutive patients with keratoconus scheduled for corneal CXL at the Hong Kong Eye Hospital and Chinese University of Hong Kong (Hong Kong SAR) in January and February 2013 were recruited. Approval from the Institutional Review Board was obtained. All patients gave a written, informed consent in accordance with the Declaration of Helsinki.

Patients with any of the following criteria were excluded from the study: age younger than 18 years old, corneal scars or opacities, previous corneal or anterior segment surgery, systemic connective tissue disease, ocular or systemic diseases that could affect epithelial healing, and pregnancy or lactation. Data obtained for analysis in this study included patient's age, gender, and slit lamp examination findings.

### 2.2. Surgical Technique

After povidone-iodine sterilization and draping of the ocular surface, a lid speculum was inserted. Epithelium was gently removed off the central 9 mm cornea. 0.1% riboflavin in 20% dextran (Medio-Cross isotonic solution; Medio-Haus Medizinprodukte, GmgH, Kiel, Germany) was then instilled every 2 minutes for 30 minutes. The corneal surface was irradiated with 18.0 mW/cm^2^ UVA light (365 nm) for 5 minutes for a total of 5.4 J/cm^2^ (CCL-HE; Peschke Meditrade, GmbH, Hunenberg, Switzerland). A silicone hydrogel bandage contact lens was inserted at the end of the procedure. Postoperative medications included 4-hourly artificial tear drops, levofloxacin drops 4 times a day until epithelization, then 0.1% dexamethasone drops 4 times a day tapered over 3 weeks.

### 2.3. Intraoperative Measurements

Intraoperative corneal thickness was measured by USP and SDOCT at 3 time points for all patients: at baseline (after insertion of speculum), after removal of corneal epithelium, and after 30 minutes of corneal saturation with riboflavin. Three patients had lid speculum on throughout the procedure, while 3 patients had their lid speculum taken out during riboflavin instillation, during which they were instructed to keep their eyes closed in between the drops. For closer monitoring of the corneal thickness of the 3 patients with lid speculum on, SDOCT images were obtained every 2 minutes (just before the instillation of riboflavin eye drop) throughout the procedure, after UVA irradiation, and after 4 hours of wearing a bandage contact lens.

#### 2.3.1. Ultrasound Pachymetry

Corneo-Gage Plus (Sonogage, Inc., Cleveland, OH) was used to measure the intraoperative central corneal thickness (CCT). The ultrasound probe was applanated against the corneal apex for measurement, the location of which was estimated by the naked eye of the surgeon. The average of 3 measurements was used for analysis. Balanced salt solution was used to wet the corneal surface when measurements could not be obtained, allegedly due to poor hydration of the corneal surface.

#### 2.3.2. Spectral-Domain Optical Coherence Tomography

**Figure 1:**
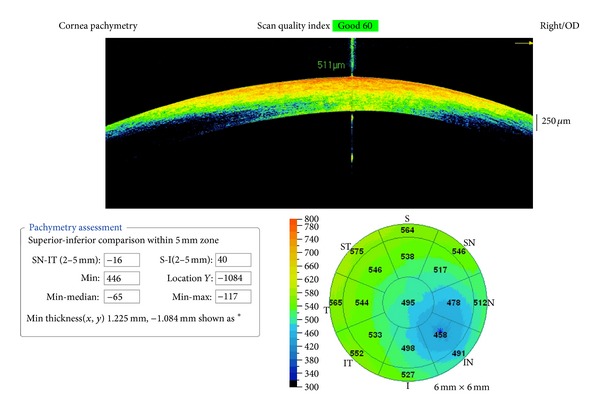
Intraoperative corneal pachymetry by spectral-domain optical coherence tomography. Cornea is divided into 16 radial zones and 1 central zone with a mean corneal thickness in each zone; the mean corneal thickness in the center is taken as the central corneal thickness (CCT). An asterisk also marks the location of the thinnest corneal thickness (TCT) on the corneal map, and its thickness and distance from the apex are presented in the “Pachymetry Assessment” table on the left lower corner.

A commercially available SDOCT machine (iVUE mounted on its iStand; Optovue Inc., Fremont, CA, USA) was used to capture images of the cornea. All intraoperative measurements were obtained under sterile conditions with patient lying supine on the operation table. The body of the SDOCT machine was wrapped in a sterile disposable plastic bag and the capturing lens protruded out of a small slit made in the bag. To keep the computer unit further away the sterile field, the capturing unit approached the patient from the temporal side with the resulting pachymetry maps rotated 90 degrees clockwise for the right eye and counter-clockwise for the left eye. Patient was asked to look at the fixation target inside the lens of the machine overhead. The SDOCT probe was centered at the corneal apex, which was identified with a bright vertical reflection. Images were captured using a foot pedal. Cornea pachymetry scan pattern was used, which captured 8 meridional cross-sectional scans (1024 axial scans per meridian) to generate a pachymetry map in the central 6.0 mm cornea (Figure 1). Three scans were performed at each time point. The pachymetry value in the central 2 mm zone was taken as the CCT, and the mean of the 3 readings was used for analysis. The location of the thinnest corneal thickness (TCT) was shown as an asterisk on the map; its value was recorded in the “Pachymetric Assessment” table on the left. The mean of three such readings was used for analysis.

### 2.4. Statistical Analysis

Statistical analysis was performed using Wilcoxon test. All analyses were done using SPSS v16.0. A *P* value ≤ 0.05 was considered to be statistically significant.

## 3. Results

Mean age of the patients was 34.5 ± 13.2 years (range, 22–58) (4 females, 2 males). All eyes had an uneventful CXL surgery. The corneal epithelium healed completely by day 3 postoperatively. None of the eyes had corneal edema detectable on slit lamp examination.

### 3.1. USP versus SDOCT Pachymetry

Table 1 shows the average pachymetric measurements at 3 time points for all 6 patients: at baseline (after insertion of speculum), after removal of corneal epithelium, and after 30 minutes of corneal saturation with riboflavin. The mean SDOCT CCT was higher than the mean USP CCT at all 3 time points but did not reach statistical significance (baseline, *P* = 0.786; after epithelial removal, *P* = 0.178; after 30 minutes of riboflavin, *P* = 0.125) (Table 1). The mean SDOCT TCT was thinner than the mean USP CCT at all 3 times points, statistically significant after epithelial removal (*P* = 0.031) and approaching statistical significance at baseline (*P* = 0.063) and after 30 minutes of riboflavin (*P* = 0.063) (Table 1).

### 3.2. SDOCT Pachymetry

**Figure 2:**
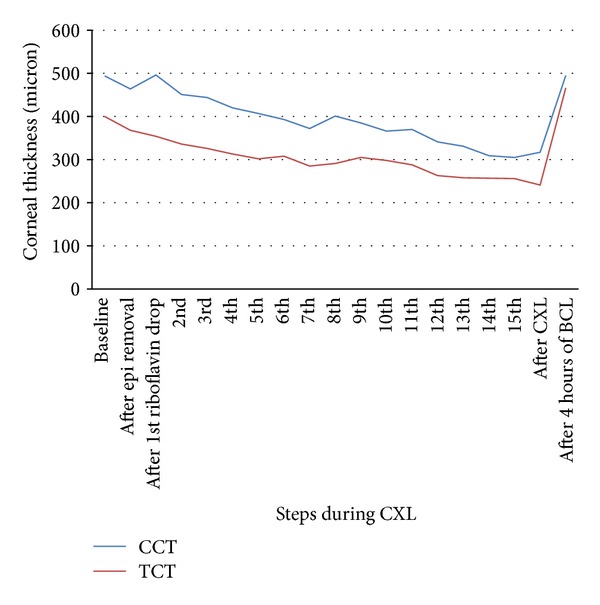
Intraoperative corneal thickness as measured by spectral-domain optical coherence tomography throughout and after the CXL procedure. Corneal thickness was measured every 2 minutes during crosslinking; CCT: central corneal thickness; TCT: thinnest corneal thickness; epi: epithelium; CXL: crosslinking; BCL: bandage contact lens.

The SDOCT showed progressive corneal thinning during CXL for all eyes. As shown in Table 1, average CCT decreased by 42.3 ± 33.4 *μ*m after epithelial debridement (*P* = 0.031) and a further 96.5 ± 64.7 *μ*m after 30 minutes of riboflavin eye drops (*P* = 0.031). There was a 28 ± 13% cumulative reduction in CCT before UVA irradiation (*P* = 0.031). The TCT decreased by 47.3 ± 28.4 *μ*m after epithelial debridement (*P* = 0.031) and a further 96.0 ± 46.1 *μ*m after 30 minutes of riboflavin eye drops (*P* = 0.031). TCT reduced by 33 ± 9% before UVA irradiation (*P* = 0.031).  Figure 2 shows the steady decline of CCT during CXL for one of the patients who had close monitoring of corneal thickness while wearing a lid speculum. The corneal thickness returned to baseline level after wearing a bandage contact lens for 4 hours for all 3 patients, with no apparent corneal edema on slit lamp examination.

### 3.3. Corneal Thickness with versus without Lid Speculum

Further analysis was performed between pachymetry of eyes with and without a lid speculum during riboflavin instillation. There was a greater reduction in CCT with the use of lid speculum with 38 ± 6% reduction in the speculum-on group compared to only 18 ± 9% reduction in the speculum-off group (*P* = 0.10) (Table 2). TCT reduced by 40 ± 5% in the speculum-on group and 26 ± 7% in the speculum-off group (*P* = 0.10) (Table 3).

## 4. Discussion

Intraoperative measurement of corneal pachymetry is an important step in CXL. However, all the studies reported so far have used USP to measure the corneal thickness during CXL, which may not be ideal due to its contact nature and inconsistent results in poorly hydrated corneas. Most importantly, USP cannot locate the thinnest point on the cornea, which is often displaced in keratoconic eyes. In the current study, we used an intraoperative noncontact SDOCT machine to measure corneal thickness during CXL. Intraoperative corneal thickness was measured successfully in all eyes in our study. Serial measurements could be obtained at frequent intervals during the surgery. While CCT measurements were comparable between SDOCT and USP, SDOCT TCT seems to be thinner than the USP CCT during the CXL procedure. Furthermore, the TCT values were well below the suggested minimum thickness requirement of 400 micron at many time points. Similar to previous studies [10–14], the corneal thickness continued to decline during the procedure.

As one of the measures to reduce the corneal thinning during corneal CXL, some authors have proposed removal of the lid speculum and keeping the eye closed during the riboflavin instillation [11, 13]. We used SDOCT to demonstrate the difference in corneal thinning with and without a lid speculum. Holopainen and Krootila [13] compared the reduction in corneal thickness during the first 30 minutes of riboflavin instillation with the eyes closed versus the latter 30 minutes of UVA irradiation while eyes were kept open by a lid speculum and found an approximately 50% lesser reduction during the first 30 minutes when eyes were closed. Kaya et al. [14] reported an 18% reduction in corneal thickness while keeping the eyes closed. Kymionis et al. [10] also reported an 18% reduction in corneal thickness before UVA irradiation, but it was not clear whether they kept the eyes open or closed during the riboflavin instillation. In our study, we showed a similar 18% reduction in CCT when eyes were closed compared to 38% when lid speculums were used. TCT was further reduced by 26% when eyes were closed versus 40% when lid speculums were used. 

These findings raise serious concern over how corneal thickness can fluctuate with a difference in the execution of the surgical steps during corneal CXL. This reduction in thickness may have been further masked by the use of USP, as precise location of the thinnest point could not be taken each time. Many authors now suggest alternative protocols to the original Dresden protocol to expand the use of CXL in thin corneas, including the use of hypotonic riboflavin [21], customised epithelial debridement [22, 23], transepithelial CXL [24], shortened irradiation duration, shortened duration of riboflavin instillation, or increased concentration of riboflavin [25]. Even riboflavin of the same concentration and tonicity, when composed with dextran as in the commercially available Medio-Cross solution or with hydroxypropyl methylcellulose as originally used by the Dresden group, can cause different degrees of corneal dehydration [15]. It will be very informative if intraoperative pachymetry could be performed with SDOCT when evaluating these different protocols to shed light on the most appropriate method for the protection of the corneal endothelium.

Another use of SDOCT not fully elaborated in this study is the study of corneal reflectivity in the high-resolution cross-sectional images of the cornea during corneal CXL. It has been used by Malhotra et al. [26] as a measure to evaluate riboflavin penetration in corneas with and without epithelial debridement. As our SDOCT machine is different from the one used by Malhotra et al. [26], we have not been able to see the absence of reflectivity at baseline that could serve as a control before riboflavin instillation as suggested. But intuitively, perhaps with some adjustment, iVUE SDOCT may also be used to assess riboflavin penetration as an indication of the effectiveness of CXL by the alternative protocols mentioned previously, in addition to providing pachymetric information.

One limitation of this study is the small sample size. Nevertheless, a difference between the two arms was already apparent, which has made the authors abandon the use of lid speculum during riboflavin instillation before culminating enough cases to reach statistical significance. Another limitation, which is beyond the scope of this study, is the inability to comment on the accuracy of the measurements made by iVUE SDOCT. It is noteworthy that the conventional “upright-sitting” counterpart (RTVue, Optovue Inc., Fremont, CA, USA) of iVUE SDOCT is a well-known device with excellent repeatability and reproducibility in measuring CCT [16–20], though measurements cannot be used interchangeably with those made by USP [18–20]. Another recent study in feline eyes has also shown that the iVUE SDOCT is capable of obtaining CCT measurements with excellent intra- and interoperator reliability [27]. Further studies will be needed to establish the accuracy of SDOCT pachymetric measurements with clinicopathological evaluations of the cornea and to study the safety and efficacy of performing CXL in corneas of the thicknesses as measured by SDOCT.

We have demonstrated the ease and usefulness of SDOCT to measure corneal thickness at different time points during corneal CXL. We believe that intraoperative SDOCT has the potential to replace USP in measuring corneal thickness during corneal CXL.

## Figures and Tables

**Table 1:** Central corneal thickness measurements with ultrasound pachymetry and spectral-domain optical coherence tomography at 3 time points before ultraviolet A irradiation.

	Mean CCT USP (range) (microns)	Mean CCT SDOCT (range) (microns)	Mean TCT SDOCT (range) (microns)
Baseline	470.0 ± 36.2 (440–510)	481.3 ± 37.8 (435–539) *P* = 0.786	428.2 ± 33.5 (395–480) *P* = 0.063
After epithelial removal	421.3 ± 25.8 (388–460)	439.0 ± 42.4 (390–498) *P* = 0.178	380.8 ± 27.7 (356–430) *P* = 0.031
After 30 minutes of riboflavin	330.0 ± 30.3 (302–369)	342.5 ± 39.4 (303–396) *P* = 0.125	284.8 ± 33.0 (249–341) *P* = 0.063

CCT: central corneal thickness; TCT: thinnest corneal thickness; USP: ultrasound pachymetry; SDOCT: spectral-domain optical coherence tomography.

Values in microns are presented as mean ± standard deviation (range).

*P* values are for comparison with ultrasonic pachymetry.

**Table 2:** Central corneal thickness measured with spectral-domain optical coherence tomography between eyes with and without lid speculum.

	With lid speculum (range) (microns)	Without lid speculum (range) (microns)	*P* value
Baseline	509.3 ± 25.7 (494–539)	453.3 ± 23.6 (435–480)	0.100
After epithelial removal	452.0 ± 53.0 (394–498)	426.0 ± 34.2 (390–458)	
After 30 minutes of riboflavin	315.7 ± 20.2 (303–339)	369.3 ± 36.3 (328–396)	
Total percentage of reduction	38 ± 6%	18 ± 9%	0.100

CCT: central corneal thickness; USP: ultrasound pachymetry; SDOCT: spectral-domain OCT.

Values in microns are presented as mean ± standard deviation (range if available).

**Table 3:** Thinnest corneal thickness measured by spectral-domain optical coherence tomography between eyes with and without lid speculum.

	With lid speculum (range) (microns)	Without lid speculum (range) (microns)	*P* value
Baseline	445.7 ± 41.2 (400–480)	410.7 ± 13.6 (395–418)	0.236
After epithelial removal	393.0 ± 32.7 (368–430)	368.7 ± 20.2 (356–392)	
After 30 minutes of riboflavin	267.7 ± 26.5 (249–298)	302.0 ± 33.8 (282–341)	
Total percentage of reduction	40 ± 5%	26 ± 7%	0.100

TCT: thinnest corneal thickness; USP: ultrasound pachymetry; SDOCT: spectral-domain optical coherence tomography.
